# Development and Characterization of Calcium Ion‐Enhanced Nanophytosomes Encapsulating Pomegranate Fruit Extract

**DOI:** 10.1002/fsn3.70032

**Published:** 2025-02-14

**Authors:** Ramesh Sedighi, Ali Rafe, Ghadir Rajabzadeh, Abbas Pardakhty

**Affiliations:** ^1^ Department of Food Physics Research Institute of Food Science and Technology (RIFST) Mashhad Iran; ^2^ Department of Nanotechnology Research Institute of Food Science and Technology (RIFST) Mashhad Iran; ^3^ Pharmaceutics Research Center, Neuropharmacology Institute Kerman University of Medical Sciences Kerman Iran

**Keywords:** calcium, encapsulation, Nano‐phytosome, phosphatidylcholine, pomegranate extract

## Abstract

Nanophytosomes (NP_S_) loaded with whole pomegranate fruit extract with peel and arils (PFE) at different levels of phosphatidylcholine (PC) were produced using a thin‐film hydration method and reinforced with calcium ions. PFE was obtained by pressing whole pomegranates, followed by mixing with PC at ratios of 1:1, 1:2, and 1:3, which then strengthens the phytosome wall by CaCl_2_ solutions (1.35 and 2.70 mM) and lyophilized to create a stable powder form. The characteristics of the NP powders, including encapsulation efficiency (EE), particle size, ζ‐potential, polydispersity index (PDI), structure, microstructure, and thermal properties, were evaluated. Additionally, the storage stability of phenolic compounds over two months was investigated. The PFE powder demonstrated appropriate characteristics for incorporation into the phytosome system, with a total phenol content of 371.19 mg GAE/g dry weight, anthocyanins at 300.68 mg/g, flavonoids at 194 mg/100 g, and an antioxidant activity of 90.98%. The highest EE was determined to be 98.53%, indicating its unique ability as a nano‐carrier. PFE‐loaded NPs showed favorable characteristics, such as low PDI values (< 0.5), smaller particle size (170 nm), and a spherical morphology. The PFE‐NP had a particle size of 128.6 nm, zeta potential of −40.15 mV, mobility of −3.15 μm cm/Vs, PDI of 0.168, and EE of 98.53%. The optimized nanoparticles remained stable for two months at 4°C, with negligible changes in particle size (~10 nm), total phenol content (TPC), and PDI of the PFE‐Nanophytosomes. All NP samples showed better stability at storage temperatures over 60 days. PEF‐NPs improved the stability of phenolic compounds while improving solubility, masking taste, and delivery to target tissues, which can be considered in future applications.

## Graphical abstract

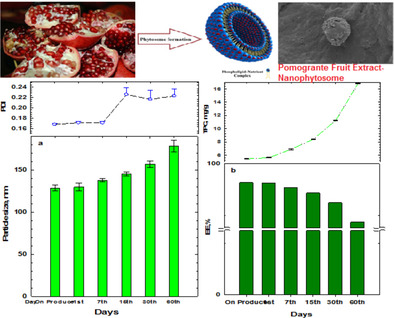

## 1 Introduction

Pomegranate ( *Punica granatum* L.) is a member of the *Punicaceae* family, believed to originate from Iran and its neighboring countries (Andishmand, Azadmard‐Damirchi, and Hamishekar 2023; Mirjalili 2016). Although pomegranate is native to Iran, it is also grown in the United States, Turkey, Spain, India, China, Tunisia, Morocco, Egypt, and even in Near and East Asia (El Barnossi, Moussaid, and Hosseini 2021). Pomegranate fruit is available in various forms, including fresh or processed pomegranate paste, seed oil, vinegar, seeds, and fruit juice (Polat, Çelik, and Kafkas 2024). The high antioxidant level and health benefits of the pomegranate and its products have fueled consumer demand. Its health effect can be attributed to polyphenolic compounds such as gallic acid, ellagic acid, and punicalagin A and B (Cheng et al. 2023). Research has shown that ellagic acid, a key phenolic compound in pomegranate, has anti‐tyrosinase, antimicrobial, anti‐inflammatory, anti‐diabetic, anti‐allergic, and anti‐mutagenic properties and may help prevent Alzheimer's disease (Suman and Bhatnagar 2019; Kyriakoudi et al. 2021; Mansoury 2019).

Pomegranate extract is a valuable source of polyphenols, which are bioactive compounds with potential health benefits. However, these polyphenols are highly susceptible to degradation when exposed to unfavorable environmental conditions such as high temperatures, oxygen, varying pH levels, and light (Kyriakoudi et al. 2021; Cao et al. 2021). Additionally, the extract faces limitations in food and pharmaceutical applications due to challenges like low stability, limited solubility, and poor bioavailability (Nouri et al. 2020; Yang et al. 2023). Furthermore, the ellagitannins in pomegranate can impart an astringent taste and may chelate metal ions (Ca, Fe, and Zn) in foods, preventing their absorption in the body. Therefore, encapsulation is a suitable method to protect the phenolic compounds, mask undesirable sensory properties, inhibit ion chelation, and ensure the gradual release of bioactive ingredients. Enhancing the stability of pomegranate fruit extract during processing, storage, or exposure to digestive enzymes can be achieved by utilizing nanophytosome carriers, which improve solubility, mask taste, and facilitate delivery to target tissues. Recent studies indicate that polyphenolic compounds in pomegranate peel have been effectively encapsulated in nanophytosomes, enhancing their bioaccessibility, storage stability, and solubility (Soltanzadeh et al. 2021; Dundar et al. 2023).

Nanophytosomes (NP) are stable complexes formed through electrostatic interactions between phospholipids and polyphenols, permitting hydrophilic bioactive substances to be trapped by phosphatidylcholine, such as lecithin (Lua et al. 2019). These NP complexes arise from the non‐covalent interactions of hydrogen bonding between bioactives and the polar functional groups of phosphatidylcholines. (Khosh manzar et al. 2021). The presence of PC, a key component of cell membranes, enhances the solubility, absorption, distribution, and bioavailability of polyphenols (Yang et al. 2020). Since NPs can enhance the solubility of lipophilic polyphenols in the aqueous system, they can facilitate the transfer of these compounds from aqueous to non‐aqueous phases, thereby releasing them into the targeted medium. Furthermore, due to the improved bioaccessibility and stability of biological materials during storage, oral and gastrointestinal bioaccessibility can also be enhanced. Therefore, several NP complexes such as cumin essential oil (Khosh manzar et al. 2021), vitamin D_3_ (Molaveisi, Shahidi‐Noghabi, and Naji‐Tabasi 2020), garlic oil (Nazari et al. 2019), bitter melon extract (Sasongko et al. 2019), rutin (Babazadeh, Ghanbarzadeh, and Hamishehkar 2017), and apigenin (Telange et al. 2017) have been developed.

In addition to the pomegranate fruit, there are by‐products such as peel, seed kernels, and parts of the mesocarp that often have low commercial value and are primarily used as animal feed. Though recent studies have demonstrated that these by‐products, particularly pomegranate seeds contain high concentrations of phenolic compounds that offer numerous health benefits (Andishmand, Azadmard‐Damirchi, and Hamishekar 2023). For instance, pomegranate peel extract has shown potential for use in developing functional foods and dietary supplements (Andishmand et al. 2024). However, there are critical challenges such as instability and low solubility, which could impede its application. Since NP systems have been used in loading polyphenolic extracts—like date vinegar, grape seed extract, blueberry extract, pine extract, and tea extracts—with enhanced stability (Yang et al. 2023), and given that research particularly focused on pomegranate peel extract using NPs is limited (Andishmand et al. 2024), the current work aims to apply the whole pomegranate fruit (peel and arils) to maximize the extraction of bioactive compounds, including phenolics, anthocyanins, and flavonoids, from both the juice and peel and improve the storage stability of pomegranate extract through nanophytosomes. This approach promotes comprehensive valorization, enhances health benefits, and supports sustainability by reducing waste. Additionally, it improves the organoleptic properties of the final product by balancing flavors and boosting antioxidant activity. Furthermore, the effect of calcium ions on improving the storage stability of nanophytosomes was also considered. The novelty of this study lies in the application of nanophytosomes based on PC reinforced with CaCl_2_ to encapsulate the whole pomegranate fruit extract, which is rich in phenolic compounds. This method promises to be cost‐effective, easy to implement on an industrial scale, allows for controlled release of phytochemicals, and improves shelf life.

## 2 Materials and Methods

### 2.1 Materials

Phosphatidylcholine (PC) (CAS Number: 97281–47‐5), ethanol 96%, Folin–Ciocalteu, DPPH (2,2‐diphenyl‐1‐picrylhydrazyl), dipotassium hydrogen phosphate, sodium nitrite, edible citric acid, aluminum chloride, NaOH, NaCl, CaCl_2_, quercetin, sodium acetate, sodium carbonate, methanol, and gallic acid were all purchased from Merck (Darmstadt, Germany). Fully ripe pomegranates ( *Punica granatum* , L.) and red grain cultivars (Raver, Kerman, Iran) were purchased from the local market.

### 2.2 Preparation of Pomegranate Fruit Extract Powder (PFE)

Pomegranate fruit extract was obtained using a press machine (Rajasekar et al. 2012). The squeezing method was utilized to extract phenolic compounds from pomegranates to gently preserve bioactive compound integrity and minimize oxidation and cell damage. While solvent extraction might yield higher concentrations, it can alter the extract's health benefits and sensory properties. By squeezing, a high‐quality extract was created which maintains the natural flavor and composition of the fruit, catering to consumer preferences for minimally processed products and enhancing its suitability for functional food applications. The whole fruit extract was then filtered through a fabric filter. The sample was centrifuged (Universal 320R, Andreas Hettich GmbH & Co. KG, Germany) at 6500 rpm for 8 min, and the supernatant was collected. One portion of the extract was stored in a freezer at −25°C for further experiments. The other portion was lyophilized by a freeze dryer (Operon, FDU‐8606, Korea) for 72 h, and the powders were preserved at −18°C in Ziploc bags.

### 2.3 Nanophytosomes Preparation

Phytosomal nanoparticles, made from phospholipids and pomegranate extract, were prepared using the thin layer hydration method, which allows for precise control over their composition and size to achieve the desired characteristics (Dundar et al. 2023; Babazadeh, Ghanbarzadeh, and Hamishehkar 2017). In brief, the preparation involves dissolving phospholipids (lecithin) in an organic solvent (ethanol) containing plant extract, vacuum evaporation, thin film formation, hydration, and phytosome suspension. Nano‐phytosome complexes containing pomegranate fruit extract (PFE‐NP) were produced using the hydration‐sonication thin layer method. The molar ratio of pomegranate fruit extract was determined based on the results of the total phenolic compounds of dry pomegranate extract. To identify the optimal nano‐phytosome formula, various molar ratios of pomegranate fruit extract and soy lecithin (1:1, 1:2, and 1:3) separately in different volumes of organic solvent (96% ethanol) (0.4%, 0.7% and 1%) were investigated. The stirring speed was maintained at 750 rpm for 1 h until the solutions became clear and homogeneous. The obtained nano‐phytosomes were labeled as PFE‐NP_1_, PFE‐NP_2_, and PFE‐NP_3_ based on the ratio of pomegranate extract to soy lecithin and then kept for 24 h at 4°C to complete interactions. The obtained mixture was transferred into a round‐bottom flask, and the solvent was removed in a rotary evaporator at a temperature of 45°C to form a thin layer around the flask. The obtained thin layer was hydrated with various volumes of deionized water to prepare 0.4%, 0.7%, and 1% of PFE. Calcium chloride solution (0, 1.35 and 2.70 mM) was added dropwise to the hydrated complex treatments to strengthen the phytosome wall. The complex was homogenized on a magnetic stirrer for 30 min at a stirring speed of 300 rpm and then further homogenized with an Ultra‐turrax (T10 Basic Ultra‐Turrax made by German company IKA) at 12,000 rpm for 10 min to ensure uniformity. Ultrasonic homogenization was performed to reduce the size of particles under ultrasonic waves with a titanium probe (7 mm) at 70% power for ten minutes and ten cycles per minute (Dundar et al. 2023; Rasaee et al. 2014). The PFE‐NP complexes were lyophilized (Operon, FDU‐8606, Korea) for 72 h and stored in a Ziploc bag in the refrigerator.

### 2.4 Anthocyanin and Flavonoid Measurements

Total phenolic content (TPC) was assessed using a modified Folin–Ciocalteu colorimetric method (Tezcan et al. 2009). A 1000 ppm solution was obtained by dissolving 50 mg of pomegranate extract powder in 50 mL of deionized water in a volumetric flask. This solution was incubated in a water bath at 30°C for 1 h, and then centrifuged at 8500 rpm for 5 min, with the supernatant used for TPC analysis. The evaluation was performed against a gallic acid calibration curve spanning 0.003–0.1 mg/mL (5 points). The linear model (y = 0.0099x + 0.011) had an R^2^ of 0.9995 (Figure S1). Samples were tested in triplicate, and results were expressed as mg gallic acid equivalent per gram of dry weight.

The total flavonoid content in plant extracts was determined using the AlCl_3_ colorimetric method. This assay involves the formation of stable complexes between the aluminum ions (Al^3+^) present in the AlCl_3_ reagent and the hydroxyl groups of flavonoids, resulting in a change in the absorption spectrum that can be measured by spectrophotometry (Tezcan et al. 2009). According to this method, 0.5 mL of diluted samples was mixed with 1.5 mL of methanol, and equal amounts of 10% aluminum chloride and 1 M potassium acetate were added to the solution. The mixture was then diluted with 2 mL distilled water, vortexed, and left at room temperature for 30 minute before measuring the absorbance at 415 nm. Quercetin was used to create a standard curve (y = 0.007x + 0.014, R^2^ = 0.99) for determining the flavonoid content, and the results were expressed in mg/L and mg/g of dry matter.

Total anthocyanin content was determined using a spectrophotometric method based on pH differences and by the method reported by Wrolstad, Durst, and Lee (2005). Initially, a stock solution of pomegranate extract powder was prepared. Following centrifugation for 10 minute at 6500 rpm, 1 mL of the Falcon supernatant was extracted and mixed with KCl buffer of pH 1 (0.25 M KCl and concentrated HCl) to reach a total volume of 10 mL. Additionally, 1 mL of extract mixed with sodium acetate buffer of pH 4.5 (containing 0.4 M of sodium acetate and concentrated acetic acid) to a volume of 10 mL was incorporated. The absorption of these samples with buffer was measured using a spectrophotometer at 510 and 700 nm. Anthocyanidin levels were quantified in terms of cyanidin 3‐glucosidase, with the total anthocyanin content in the extract expressed in milligrams of cyanidin‐3‐glycoside per gram of dry powder according to the following equations (1), (2):

(1) $$∆A={\left(A_{510}-A_{700}\right)}_{\mathrm{at}\;\mathrm{pH}\;1}-{\left(A_{510}-A_{700}\right)}_{\mathrm{at}\;\mathrm{pH}\;4.5}$$

(2) $$C\left[\frac{\mathrm{mg}}{L}\right]=\frac{\left(∆A\times M_{w}\times D_{f}\times 1000\right)}{\left(\varepsilon \times L\right)}$$

Where ∆*A* is the absorption difference, *M*_*W*_ is the molecular weight of dominant anthocyanin (449.2), *D*_*F*_ is the dilution factor, *ε* is the molar absorption of dominant anthocyanin, and *L* is the cell length in cm.

### 2.5 Assessing and Quantifying Antioxidant Activity

The antioxidant capacity of pomegranate extract was evaluated using the DPPH method with the free radical 2,2‐diphenyl‐1‐picrylhydrazyl (DPPH) (Tezcan et al. 2009). A 0.008% DPPH solution was initially prepared for this analysis (Elfalleh et al. 2012). A stock solution of extract (1000 ppm) along with various solutions was prepared, which was protected from light by foil. In the control sample, 0.5 mL of methanol replaced the extract, while 0.5 mL of the extract was combined with 1.5 mL of DPPH solution. They were then vortexed and incubated in adark place for 30 min prior to measure the absorbance. Calibration of the spectrophotometer was performed with 85% methanol.

### 2.6 Characterization of PFE‐NP

#### 2.6.1 Analysis of Particle Size, Polydispersity, and ζ‐ Potential

Particle size, polydispersity index (PDI), and ζ‐potential were analyzed using a dynamic light scattering (DLS) particle size analyzer (Nano ZS, ZEN3600, Malvern Instruments, UK). To minimize multiple scattering effects, samples were diluted 10‐fold with deionized water. Samples were then placed in a quartz cuvette and assessed at a 90^o^ scattering angle. Mean particle size was determined using Average‐Z, while nanoparticle uniformity and aggregation tendencies were evaluated using the PDI factor, which indicates overall particle consistency and behavior (Sze et al. 2003). ζ‐potential and electrophoretic mobility were determined to check the surface charge and movement of charged particles of nano‐phytosomes (Barbosa, Abdelsadig, and Conway 2019).

#### 2.6.2 Encapsulation Efficiency and Loading Capacity

An indirect method was used to determine the encapsulation efficiency (EE) of pomegranate extract inside the nanophytosomes. For this purpose, appropriate concentrations of pomegranate extract were prepared with ethanol, and the absorption rate of the sample at the maximum absorption was read based on the data obtained from scanning by UV–vis spectrophotometer, and then the calibration curve was drawn, and its equation was determined (Babazadeh, Ghanbarzadeh, and Hamishehkar 2017). The encapsulation efficiency, which is denoted as EE%, of PFE when using soy lecithin as a carrier was determined through the application of Dündar's method, albeit with some minor modifications to adapt the procedure to our specific requirements (Dundar et al. 2023). Initially, the total phenolic content (TPC) of pomegranate fruit extract was determined before and after the formation of a nano‐phytosome. For this purpose, 1 g of PFE‐NP extract and sample was mixed with 10 mL of ethanol/water (70/30 v/v) in a shaking water bath at 25°C for 2 h. Then the mixtures were centrifuged, and the supernatant was used for TPC analysis. EE was determined by the equation (3):

(3) $$\mathrm{EE}\left(\%\right)=\frac{{\mathrm{TPC}}_{\mathrm{PFE}}-{\mathrm{TPC}}_{\mathrm{NC}}}{{\mathrm{TPC}}_{\mathrm{PFE}}}\times 100$$

*Where* TPC_PFE_ is the total phenolic content of pomegranate fruit extract and TPC_NC_ is the total phenolic content of nano‐phytosome supernatant. Loading capacity (LC %) is calculated by dividing the mass of TPC in phytosome by the mass of phytosome (Dundar et al. 2023) (Khosh Manzar et al. 2021).

#### 2.6.3 PFE‐NP Powder Physical Properties

The moisture content of the PFE‐NP powders was assessed in triplicates at 105°C using an infrared analyzer (Ohaus, MB45), (Dadi et al. 2020). The water activity (a_w_) of the pomegranate fruit extract nanoparticles (PFE‐NP) was determined at 25°C using a water activity meter (Novasina, Ag/Lab Master, Sweden). Calibration of the water activity meter was carried out with ultrapure water and a 2.33 mol/kg NaCl solution (0.920 aw) (Szulc and Lenart 2013).

The water solubility index (WSI) and swelling power of PFE‐NP powders were evaluated using a modified Li method (Li et al. 2018). Briefly, 0.15 g of PFE‐NP powders were mixed with 10 mL of distilled water in a Falcon tube, heated at 85°C for 30 minute, and centrifuged at 8000 rpm for 10 minute. The supernatant was dried at 105°C for 12 hour to calculate WSI (Equation 4), while the water binding capacity of the pellet was determined from the weight of the remaining paste per gram of dry PFE‐NP powder.

(4) $$\mathrm{WSI}=\frac{M_{\text{supernatant}}}{M_{\text{Powder}}}\times 100$$

The color profile (L*, a*, b*) of PFE‐NP was analyzed using a colorimeter (PCE, CSM3, UK). The device was calibrated with a white disk. PFE‐NP powder in a Petri dish was measured at five locations in darkness (Dundar et al. 2023; Dundar et al. 2023). Total color difference (ΔE) was calculated using (Equation 5):

(5) $$∆E=\sqrt{{\left(∆L^{*}\right)}^{2}+{\left(∆{\mathrm{La}}^{*}\right)}^{2}+{\left(∆b^{*}\right)}^{2}}$$

Bulk density (ρb) and tapped density (ρt) of PFE‐NP powders were measured using a 10 mL cylinder. Bulk density indicates the mass‐to‐volume ratio, while tapped density was measured after 125 manual taps. Hausner's ratio (HR) and Carr's index (CI) were calculated from ρb and ρt (Dundar et al. 2023).

(6) $$\mathrm{HR}=\frac{\rho_{t}}{\rho_{b}}$$

(7) $$\mathrm{CI}=\frac{\rho_{t}-\rho_{b}}{\rho_{b}}\times 100$$

Szulc and Lenart (2013) established a classification system for powder cohesion using the Hausner Ratio (HR), designating values below 1.2 as low cohesion, those ranging from 1.2 to 1.4 as medium cohesion, and values exceeding 1.4 as high cohesion. Additionally, they assessed powder flowability based on the Carr Index (CI), categorizing it as very good for CI values under 15%, good for values between 15% and 20%, fair for values from 20% to 35%, bad for values from 35% to 45%, and very bad for values above 45%.

### 2.7 Microstructural Attributes of PFE‐NP

A scanning electron microscope (SEM) was employed to examine the microstructure of PFE‐NP. Samples were gold‐coated using a vacuum coating device. High‐speed electrons at 15 kW voltages were directed at the samples, producing an image based on the returning electron beam. Subsequently, imaging and analysis of the samples were conducted using a non‐exciting SEM (KYKY‐EM3200; KYKY Technology Development Ltd., Beijing, China) at 25 KV with 20,000× magnification.

### 2.8 Chemical Structure of PFE‐NP

The detection of structural changes and functional groups in PFE‐NPs and its individual components (pomegranate fruit extract powder, soy lecithin, CaCL_2_) was performed by FT‐IR spectroscopy (Bruker Alpha II, UK) within wavenumbers of 4000–400 cm^−1^. Through stoichiometric interactions, the formation of chemical bonds between the −OH groups of bioactive materials and phospholipids was investigated. PFE, LC, CaCL_2_ and PFE‐NP spectra were analyzed with IRPAL software (Babazadeh, Ghanbarzadeh, and Hamishehkar 2017).

### 2.9 X‐Ray Diffraction of PFE‐NP

Thermal analysis of the nano‐phytosome structure was performed by an XRD device (D8 Advance, Bruker, Germany). The beam diffraction was scanned in the angle range (2θ) from 5° to 90° (Hasanvand and Rafe 2018; Nazari et al. 2019).

### 2.10 Thermal Properties of PFE‐NP

Thermal properties of nano‐phytosome with pomegranate fruit extract were assessed using a DSC (Netzsch‐Gerätebau GmbH—STA 409 PC Luxx Simultaneous thermal analyzer). Indium and zinc served for temperature and heat flow calibration, while a 50‐μL aluminum container was selected as a reference. Approximately 5 mg of the sample was moved into an aluminum container. Heating and cooling proceeded at 10°C per minute, within a temperature range of 20°C to 400°C. Samples underwent heating from 20°C to 400°C and were then held at this temperature for 1 min (Telange et al. 2017).

### 2.11 Physical Stability of PFE‐NP

To evaluate the stability of nano‐phytosome formulations, particle size changes were examined for 60 days at 4°C (Pezeshki, Hamishehkar, and Ghanbarzadeh 2019). Moreover, the physical stability of the nano‐phytosome structure containing pomegranate fruit extract was assessed over time by storing the solutions in 15 mL flasks at 4°C. The stability and encapsulation efficiency of the pomegranate fruit extract in the nano‐phytosome were determined on the 1st, 7th, 15th, 30th, and 60th days post‐production by measuring absorbance in a spectrophotometer (Molaveisi, Shahidi‐Noghabi, and Naji‐Tabasi 2020). The stability was calculated using the following equation (Equation 8):

(8) $$\text{Nanophytosome Stability}=\frac{\text{Amount of total phenol in nano}-\text{phytosome remaining}\;}{\text{Amount of initial encapsulated total phenol}}\times 100$$

### 2.12 Statistical Assessment

All tests were conducted in a completely randomized factorial design with three replications. The analysis of the data was carried out through a one‐way ANOVA with a significance threshold set at *p* < 0.05. Means were compared using Duncan's multi‐range test and SPSS version 16 software. The response surface method (RSM) was employed during the optimization phases.

## 3 Results and Interpretation

### 3.1 Anthocyanin and Antioxidant Properties

Anthocyanin and antioxidant properties of PFE were evaluated, and it was found that the total anthocyanin, flavonoid, and phenolic content of pomegranate fruit extract were 300.68 ± 7.56 mg/g, 194.97 ± 0.01 mg/100 g, and 371.19 ± 8.12 mg GAE/g dry weight, respectively. These values were higher than that of pomegranate seed or peel (Dundar et al. 2023). By increasing Ca^2+^concentration, DPPH was increased from 86.90 ± 0.05, 87.05 ± 0.01 to 87.45 ± 0.01 for the PEF‐NP complexes. However, it was further increased when the PEF/PC ratio was increased as the highest DPPH was achieved 88.48 ± 0.02, 88.95 ± 0.01, and 90.92 ± 0.02, respectively. The total phenolic compound also showed the same trend, whereas the PEF/PC ratio increased, and TPC was enhanced from 263.99 ± 20.18, 262.77 ± 6.90, and 321.97 ± 5.92, in the absence of calcium chloride. By increasing calcium chloride, the TPC was increased for PEF‐NP_3_ to 355.71 ± 1.07 and 365.72 ± 1.27 at 1.35 and 2.70 mM CaCl_2_, respectively.

### 3.2 PFE‐NP Powder Properties

According to the preliminary experiments, NP complexes at different PFE to PC ratios were selected as PFE‐NP_1_, PFE‐NP_2_, and PFE‐NP_3_. The physicochemical properties of PEF and PEF‐NP powders as affected by PFE/PC ratios and calcium chloride concentrations are summarized in Tables 1, 2. As it can be seen by increasing the PEF to PC ratio, moisture content and water activity did not statistically change in the samples in the presence or lack of calcium ions, however by increasing CaCl_2_, moisture content and a_w_ decreased. In contrast, by increasing PC, L* and b* values in nano‐phytosome significantly increased, which caused a brighter and yellowish powder. In contrast, the a* value decreased by lecithin content, which may be attributed to the higher moisture content. Indeed, increased levels of unsaturated fatty acids from PC in the nano‐phytosome potentially lead to oxidation and color changes. The calcium chloride also showed the same trend in the color properties of the nanophytosomes. Moreover, the brightness of the nano‐phytosome may also be associated with the higher L* value in PC. Due to the high solubility in the water of PC, the WSI of nano‐phytosomes significantly increased. Increasing PC in PFE‐NP_2_ and PFE‐NP_3_ induced a major reduction in a_w_ and high WSI was obtained. The flowability of nano‐phytosome powder is typically evaluated using the Hausner ratio (HR) to measure inter‐particle friction and the Carr index (CI) to assess the strength and stability of potential powder arches or bridges (Kushwaha et al. 2020). PFE‐NP at different PC ratios showed a reduction in HR and CI results. According to Szulc and Lenart (2013), PFE‐NP powders revealed intermediate cohesiveness and fair flowability due to the HR results of 1.16–1.22 and CI in the range of 15.3%–22.2%, respectively. Furthermore, the calcium ion did not significantly influence the HR and CI results of nano‐phytosomes (*p* < 0.05). These results indicated that increasing the PC ratio and calcium chloride concentration in nanophytosomes decreased the flow properties of the powder due to the high hygroscopicity of phosphatidylcholine and calcium chloride. Similar findings in the physical properties of NP complexes of peeled pomegranate have been reported recently (Dundar et al. 2023).

**TABLE 1:** Characteristics of pomegranate fruit extract (PFE) and Nano‐phytosome powders at different PFE to PC ratios (1:1, 1:2, 1:3). No CaCl_2_ was used (control sample)*.

Properties	PFE	PEF‐NP_1_	PEF‐NP_2_	PEF‐NP_3_
Moisture, %	5.26 ± 0.08	3.45 ± 0.16^a^	3.65 ± 0.13^a^	3.32 ± 0.11^a^
a_w_, −	0.38 ± 0.03	0.46 ± 0.25^a^	0.42 ± 0.24^b^	0.41 ± 0.17^b^
L*	7.44 ± 1.31	62.13 ± 2.17^c^	65.32 ± 2.05^b^	69.35 ± 1.19^a^
a*	5.64 ± 2.36	18.48 ± 1.05^a^	14.26 ± 1.24^b^	14.24 ± 1.74^b^
b*	1.15 ± 0.95	2.35 ± 0.16^b^	2.58 ± 0.15 ^a^	2.55 ± 0.69^a^
ρ_b_, g/cm^3^	0.082 ± 0.011	0.034 ± 0.121^c^	0.042 ± 0.152^b^	0.053 ± 0.093^a^
ρ_t_, g/cm^3^	0.101 ± 0.027	0.405 ± 0.153^c^	0.050 ± 0.132^b^	0.062 ± 0.127^a^
HR, −	1.234 ± 0.001	1.185 ± 0.004^a^	1.185 ± 0.003 ^a^	1.163 ± 0.001^b^
CI, %	23.35 ± 0.14	17.79 ± 4.17^b^	18.22 ± 3.45 ^a^	16.29 ± 1.11^c^
WSI	85.36 ± 1.02	88.67 ± 1.02^b^	87.88 ± 2.13^c^	89.47 ± 2.35^a^
WBS	72.45 ± 2.87	134.66 ± 2.32^a^	149.06 ± 1.58 ^a^	148.58 ± 3.76^b^

*Note:* Data are provided in means±SD, and alphabetic symbols indicating the significant difference between nanophytosomes (*p* < 0.05).

Abbreviations: a_w_, water activity; CI, Carr index; HR, Hausner ratio; PEF‐NP, pomegranate fruit extract‐nanophytosomes; PFE, pomegranate fruit extract; WBC, water binding capacity; WSI, water solubility index.

**TABLE 2:** Impact of CaCl_2_ concentrations on the physical properties of nanophytosomes of pomegranate fruit extract (PFE) at different PFE/PC ratios 1:1, 1:2, and 1:3*.

Properties	[Ca] = 1.35 mM			[Ca] = 2.70 mM		
	PEF‐NP_1_	PEF‐NP_2_	PEF‐NP_3_	PEF‐NP_1_	PEF‐NP_2_	PEF‐NP_3_
Moisture, %	3.42 ± 0.22^a^	3.28 ± 0.32^b^	2.65 ± 0.25^c^	3.48 ± 0.28^a^	2.58 ± 0.29^b^	2.15 ± 0.21^c^
a_w_, −	0.45 ± 0.54^a^	0.38 ± 0.35^b^	0.40 ± 0.27^b^	0.45 ± 0.13^a^	0.39 ± 0.14^b^	0.36 ± 0.12^c^
L*	65.85 ± 2.21^c^	68.56 ± 3.09^b^	70.48 ± 3.01^a^	68.25 ± 3.16^b^	70.63 ± 3.09^a^	71.33 ± 3.09^a^
a*	18.41 ± 2.15^a^	12.86 ± 1.02^c^	14.02 ± 1.55^b^	18.36 ± 1.23^a^	13.29 ± 1.57^c^	15.17 ± 1.80^b^
b*	2.16 ± 0.26^b^	2.72 ± 0.12^a^	2.65 ± 0.14^a^	2.65 ± 1.02^a^	2.75 ± 1.48^a^	1.65 ± 0.36^b^
ρ_b_, g/cm^3^	0.035 ± 0.13^c^	0.045 ± 0.08^b^	0.053 ± 0.01^a^	0.036 ± 0.08^c^	0.046 ± 0.17^b^	0.057 ± 0.15^a^
ρ_t_, g/cm^3^	0.042 ± 0.42^c^	0.055 ± 0.35^b^	0.062 ± 0.12^a^	0.042 ± 0.12^c^	0.054 ± 0.15^b^	0.066 ± 0.02^a^
HR, −	1.20 ± 0.02^a^	1.22 ± 0.04^a^	1.16 ± 0.01^b^	1.17 ± 0.04^a^	1.16 ± 0.04^a^	1.15 ± 0.03^a^
CI, %	20.48 ± 2.26^a^	21.95 ± 3.24^a^	16.29 ± 1.10^b^	16.91 ± 4.63^a^	15.98 ± 4.25^b^	15.03 ± 3.24^b^
WSI	87.34 ± 2.19^a^	88.69 ± 2.51^a^	89.71 ± 1.21^a^	85.98 ± 1.45^c^	89.02 ± 2.13^b^	94.51 ± 1.09^a^
WBS	136.26 ± 2.12^b^	152.03 ± 1.38^a^	138.58 ± 2.36^b^	138.26 ± 1.29^b^	147.04 ± 2.89^a^	134.44 ± 1.66^b^

*Note:* Data are provided in means±SD, and alphabetic symbols indicating the significant difference between nanophytosomes (*p* < 0.05).

Abbreviations: a_w_, water activity; CI, Carr index; HR, Hausner ratio; PEF‐NP, pomegranate fruit extract‐nanophytosomes; PFE, pomegranate fruit extract; WBC, water binding capacity; WSI, water solubility index.

### 3.3 Characterization of PFE‐Loaded Nano‐Phytosomes

**FIGURE 1:**
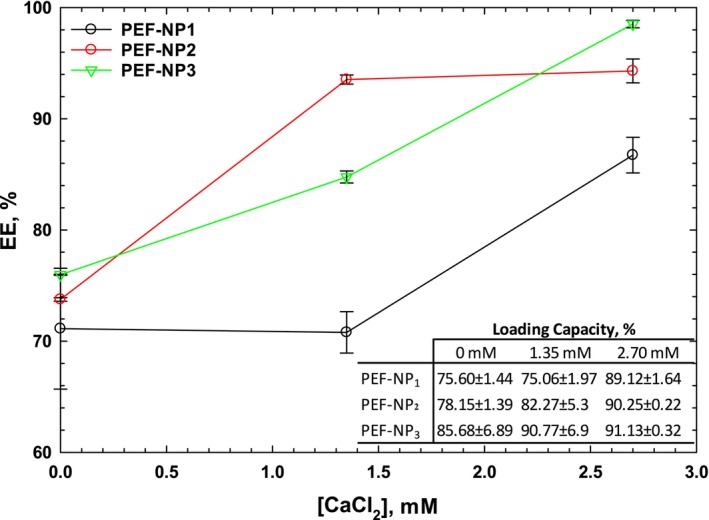
Effect of PC/PEF ratio and Ca^2+^concentration on the encapsulation efficiency (EE) and loading capacity (LC) of PFE‐NP complexes.

The effect of the PC to PEF ratio and calcium ion concentration on the EE and LC of PFE‐NP complexes is evaluated and given in Figure 1. However, EE and LC of PFE‐NP complexes were not significantly affected by the PFE/PC ratio in the control samples, while by increasing the calcium concentration, the PFE/PC ratio exhibited a significant effect on these parameters, and higher EE and LC were achieved. Although a reduction in the PFE/PC ratio slightly increased the EE while decreasing the LC. The highest EE was obtained for PFE‐NP3 (93.70%), which may be related to the sufficient amount of lecithin making more interactions between PFE and the polar group of (PC). The obtained results indicated that PFE‐NP successfully occurred and showed unique and outstanding capacity as a nano‐carrier of PFE. The loading capacities of PFE‐NP_1_, PFE‐NP_2_ and PFE‐NP_3_ at 2.7 mM of CaCl_2_ were 89.12%, 90.25% and 91.13%, respectively, which were higher than the nano‐liposomal structure of curcumin with LC less than 10% (Gomez‐Mascaraque et al.). The higher LC values of the nanophytosomes may be attributed to the PC, which has inner and outer polar groups for PFE.

**FIGURE 2:**
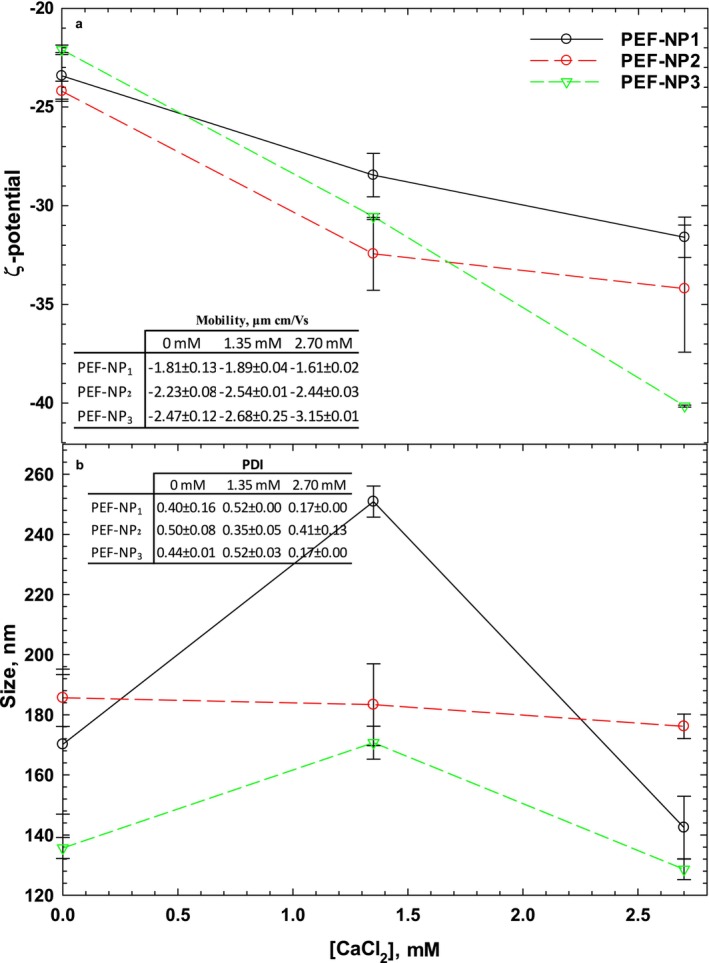
Effect of PC/PEF ratio and Ca^2+^concentration on ζ‐potential (mv) and electrophoretic mobility (μm cm/Vs) (a), and particle size and PDI (b) of PFE‐NP complexes.

Particle size, ζ‐ potential, mobility, and PDI are critical factors for evaluating the consistency, biodistribution, targeting capability, and robustness of nanocarrier systems such as nanophytosomes. The average particle sizes and PDI of PFE‐NP at different PC/PEF ratios and calcium ion concentrations are provided in Figure 2b. All the samples showed D_z_ (mean of particle size) in the nanoscale, although by encapsulating the PFE, the size was increased. Although calcium concentration did not show any significant effect on the particle size of PEF‐NP_2_, it has improved the particle size at 1.35 mM [Ca] and reduced the size at 2.70 mM [Ca] for PEF‐NP_1_ and PEF‐NP_3_. The highest value of particle size (250 nm) was achieved at 1.35 mM [Ca] for PEF‐NP_1_. Whereas the PC/PEF ratio did not exhibit any significant effect on the particle size and PDI of nanophytosomes (*p* < 0.05). The results clearly show that PFE‐nanophytosomes were successfully produced at the nanoscale, which could enhance therapeutic efficacy and gastrointestinal digestibility; this will be further explored in in vitro bioaccessibility. The particle size of vitamin D3‐loaded nanophytosomes varied from 134.9 to 400.31 nm (Molaveisi, Shahidi‐Noghabi, and Naji‐Tabasi 2020) & (Xie et al. 2023). In various targeted drug delivery systems in food engineering and modern medicine, Tan, Liu, Chen, Wu, Wang, Yin, et al. (2012) reported that the particle size of evodiamine‐phospholipid complexes was 241.10 nm, crucial for the stable release of active substances in oral environments. In another study, the particle size of NP complexes loaded with garlic essential oil was determined from 115 to 161 nm (Nazari et al. 2019). The average particle size of PFE‐NP complexes at 1.35 mM [Ca] was determined as 250.10, 183.35 and 170.72 nm for PFE‐NP_1_, PFE‐NP_2_ and PFE‐NP_3_, respectively, and these results were by the aforementioned nanophytosomes. The NP complexes showed storage stability and in vitro bioaccessibility, with average particle sizes reflected in PDI values. Values below 0.5 indicate better stability; all samples had PDI < 0.5, demonstrating homogeneity and a narrow distribution. PFE‐NP_1_ and PFE‐NP_3_had similar PDI values, while PFE‐NP_2_'s values were significantly higher, potentially representing the electrokinetic characteristics of the surface of charged particles in the colloidal system and its effect on the absorption or distance of charged particles from each other. Electrophoretic mobility refers to the amount of movement of charged particles in the colloidal system, which is usually measured as the rate of movement of particles per unit of electric field or ζ‐Potential. Mobility indicates the ability of charged particles to move in the colloidal system. Generally, higher mobility values indicate better mobility and greater stability of charged particles in the system. The electrostatic charge of particles is essential for the stability of colloids. In particle chemistry, this electrical potential is represented by the Greek letter zeta (ζ). This potential surrounds particles, cells, and other solid surfaces within the electrolyte. The effect of the PC/PEF ratio and calcium ion on the ζ‐potential and mobility was given in Figure 2a. When there is no calcium ion in the system, the highest ζ‐potential value (−23.4 mV) was observed for PFE‐NP_1_, and the lowest one was PFE‐NP_3_ (−31.6 mV). PFE‐NP_3_ showed significantly lower ζ‐potential values than PFE‐NP_1_ and PFE‐NP_2_, which had statistically similar ζ‐potential values. The negative zeta potential results of the NP complexes were probably due to the negative charge of the lecithin and PFE. The higher surface charge of PFE led to an increase in ζ‐potential values of NPs. Nazari et al. (2019) The ζ‐potential values for garlic essential oil‐loaded nanophytosomes were reported to range from −3.83 to −12.36 mV. This variation may be attributed to the interaction between the negative charge of lecithin and the positive charge of the sulfur groups in allicin, which is the main component of garlic essential oil. Furthermore, it is recommended that future studies explore the use of PFE‐loaded nanoparticle complexes in emulsion systems. The presence of negative electrical charges may enhance the stability of these emulsions. The electrophoretic mobility of PFE‐NP_1_ (−1.81) was significantly higher than other nanophytosomes (−2.23 and − 2.47), which is also in agreement with the ζ‐potential. By increasing the [Ca], both ζ‐potential and electrophoretic mobility were decreased, indicating the effect of calcium ions on the stability of the nanophytosomes.

It was found that the effectiveness of 9 apigenin‐phospholipid phytosome treatments ranged from 67%–94%, which was consistent with the range of phytosome treatments in this study (70%–98%) (Telange et al. 2017). Soy lecithin, a lipophilic compound, binds to the hydrophobic tails of PFE and helps bind hydrophilic and hydrophobic polyphenols. Jagtap et al. mapped a particle size range of 187–1045 nm for nano‐phytosome treatments, while Babu et al. reported Sizes within 100–200 nm, with an optimum size of 145.6 nm (Jagtap et al. 2023); (Babu and Shanmugasundaram 2023). It has been found that empty nano‐phytosomes showed larger particle sizes compared to particles containing pomegranate peel extracts (Andishmand et al. 2024). The inclusion of PFE extracts probably increases the electrostatic repulsion between spherical nano‐phytosomes minimizes PDI and prevents aggregation. A higher ζ‐potential indicated a stable phytosome structure due to the interaction between the pomegranate extract and the polar group of PC lecithin.

### 3.4 Nano‐Phytosome Structural Stability

According to the previous results, the highest EE and LC were obtained for PEF‐NP_3_ at 2.7 mM CaCl_2_. Under these conditions, more stability and ζ‐potential as well as less particle size and PDI were achieved. Furthermore, the highest amounts of anthocyanin and antioxidants were entrapped at PEF‐NP_3_ at 2.7 mM CaCl_2_. Therefore, we examined the storage stability of PFE in two months and then evaluated nano‐phytosomes' chemical structure, microstructure, and thermal properties.

**FIGURE 3:**
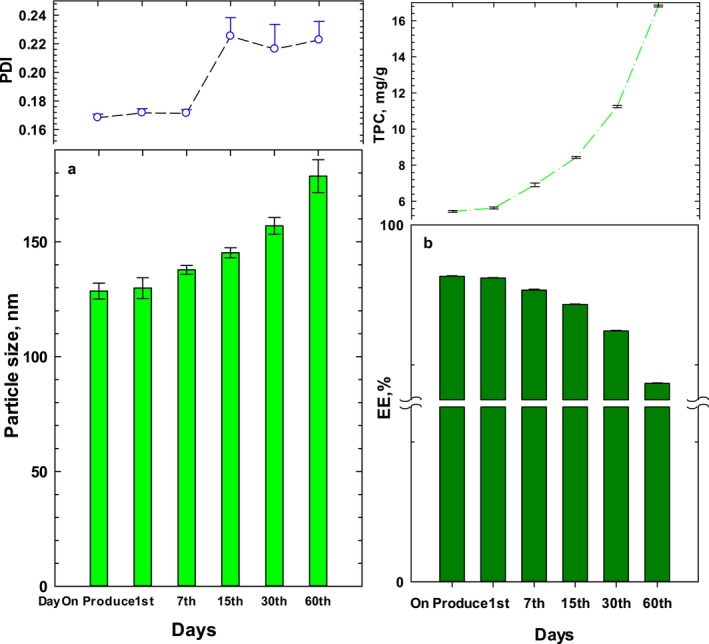
Evaluating shelf life on nano‐phytosome (PFE‐NP_3_) during two months: (a) changes in the particle size and PDI, (b) in encapsulation efficiency and total phenolic compounds.

The storage stability of PFE‐loaded NP complexes was evaluated based on particle size and PDI (Figure 3a), as well as EE and TPC compounds (Figure 3b). It can be concluded that the stability of PFE‐NP complexes was significantly improved compared to crude PFE, indicating that lecithin‐based nanophytosomes offer a protective effect against the degradation of phenolic compounds. Generally, the microencapsulation process helps preserve phenolic compounds in PFE, and the reduction in total phenolic content (TPC) loss was also noted earlier by Çam et al. 2014). The loss rate at 25°C was highest, reaching maximum TPC loss on the 60th day of storage. The production of PFE‐loaded NP complexes significantly reduced the TPC loss during all storage periods. This result may be related to the hydrogen bond formation between hydroxyl groups “the bioactive compounds” and the polar group of lecithin, which is confirmed by the FTIR results in the next sections. Nano Phytosomes containing vitamin D3 demonstrated greater stability than free vitamin D3 over a storage period of 90 days (Molaveisi, Shahidi‐Noghabi, and Naji‐Tabasi 2020). It was found that increasing the ratio of rutin to PC did not significantly affect the size of the nano‐phytosomes (Babazadeh, Ghanbarzadeh, and Hamishehkar 2017). The structural stability of phytosomes over one month at +4°C has been surveyed and out of six formulas tested, only two remained stable, with no significant changes in particle size observed (Andishmand et al. 2024). In a separate study, the physical stability of phytosomes was examined for up to one month, with the optimal formula showing a particle size of 187.1 nm (Jagtap et al. 2023). The current study evaluated the structural stability of phytosomes over two months, analyzing particle size, PDI, and encapsulation efficiency.

The TPC loss in NP samples increased significantly as the PFE ratio decreased. Among all storage conditions, PFE‐NP_1_ exhibited the highest losses. This is likely due to the high unsaturated fatty acid content (80%) in soybean lecithin, which accelerates oxidative degradation (Hager, De Paoli, Ihlo, Farach, and Poole 1993). Additionally, the reduction in particle size of NP complexes as the PFE ratio decreases may contribute to the loss of total phenolic content (TPC) during storage. This is likely due to the increased surface exposing the compounds to oxygen. Consequently, it can be concluded that nano‐phytosome technology, particularly with the NP3 formula, is effective in protecting the phenolic compounds of PFE from environmental conditions.

### 3.5 Structural Morphology (SEM)

**FIGURE 4:**
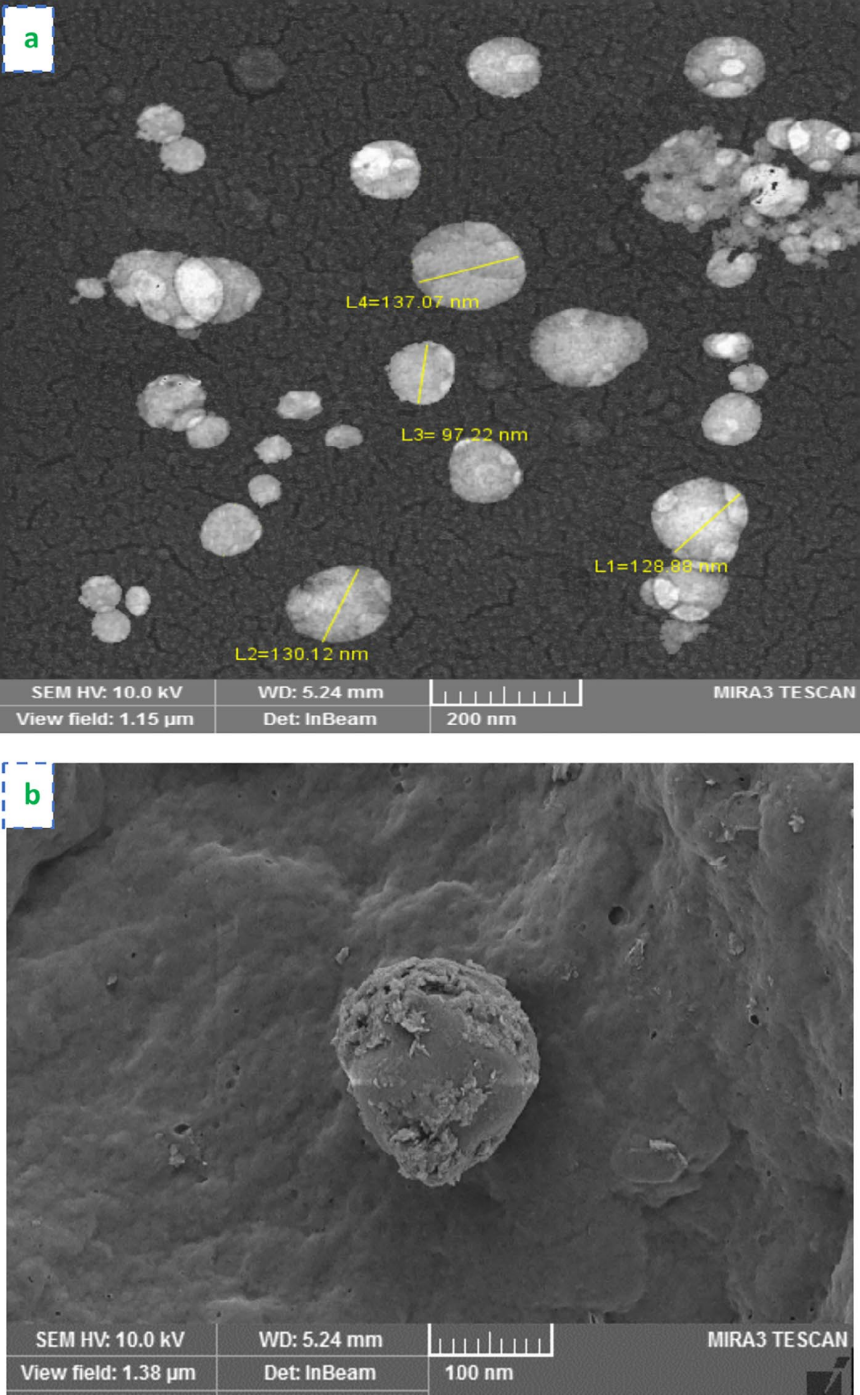
Microstructural characterization of PEF‐NP3 after production (a) and storage for 2 months (b). Small arrows show the spherical shapes of nanophytosomes.

PFE particles were spherical, but after complexation with phosphatidylcholine (PC), they transformed into nanophytosomes with a fluffy and porous surface. The SEM images of nanophytosomes are provided in Figure 4 a, b. This transformation indicates a strong interaction between PFE and PC. The spherical morphology at the nanoscale offers several advantages, including increased surface area, enhanced solubility, improved dispersion, optimal drug release, and greater bioactivity. These changes collectively enhance the overall efficacy of the formulation (Telange et al. 2017; Yang et al. 2023). PFE‐NP3 exhibited a dense and compact structure, likely due to the high ratio of PC. Scanning electron microscopy (SEM) analysis indicated that the optimal PFE/PC ratio was 1:3, leading to the best nanophytosome structure and the highest entrapment efficiency (EE) values for PFE‐NP3. A similar spherical nanophytosome structure was reported by Khosh Manzar et al. (2021) for cumin essential oil. Additionally, another study identified an optimal vitamin D3/PC ratio of 0.19, which also produced spherical nano‐phytosome complexes (Molaveisi, Shahidi‐Noghabi, and Naji‐Tabasi 2020).

### 3.6 FTIR Characteristics of PEF‐NP Complexes

FTIR analysis is a powerful tool for identifying functional groups and structural modifications in materials by examining specific absorption frequencies. This technique provides valuable insights into the chemical interactions among components and yields rapid results without the need for toxic reagents or extensive sample preparation. In the context of nano‐phytosome formation, bioactive compounds are encapsulated by phosphatidylcholine (PC) through interactions that establish chemical bonds between the hydroxyl (OH) groups of the bioactive compounds and the phospholipids (lecithin) (Patel et al. 2014).

**FIGURE 5:**
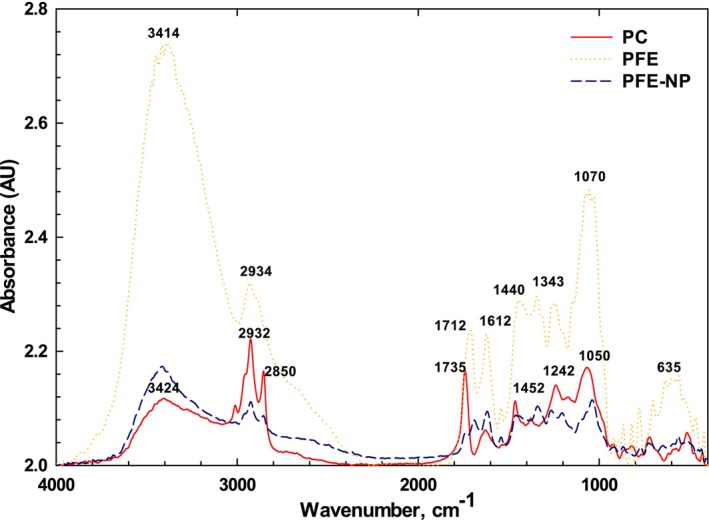
FTIR spectra of PC, PEF, and PEF‐NP_3_.

The FTIR spectrum of the PFE, PC, and NP complex reveals several significant features (Figure 5). In the PFE spectrum, broad peaks at 3414 cm^−1^ and 2934 cm^−1^ correspond to the stretching vibrations of hydroxyl and aliphatic C—H groups. A sharp peak at 1712 cm^−1^ indicates the presence of C=O groups from carboxylic acids as well as C=O groups from ketones and aldehydes. Additional peaks at 1612 cm^−1^ and 1343 cm^−1^ are associated with C=O and C=C stretching vibrations, along with aromatic ring skeletal vibrations. Furthermore, a band at 1070 cm^−1^ is related to C—O stretching and OH deformation of alcohols, while a peak at 635 cm^−1^ is linked to the O—H deformation of organic acids (Saadat et al. 2021).

The FTIR spectrum of PC is characterized by several notable absorption bands: 3424 cm^−1^ (stretching of the free hydroxyl group in alcoholic esters), 2932 cm^−1^ and 2850 cm^−1^ (C—H stretching vibrations from the long fatty acid chain), 1735 cm^−1^ (C=O stretching vibration), 1452 cm^−1^ (C—H stretching vibration of the methyl group), 1242 cm^−1^ (P=O stretching vibration), and 1050 cm^−1^ (P—O—C stretching vibration). These absorption bands provide critical information regarding the chemical structure of PC (Nazari et al. 2019) (Figure 5).

For PFE‐loaded nanophytosomes, the absorbance peak at 3424 cm^−1^ indicates an increase in hydrogen bonding due to nanoparticle formation. The FTIR results suggest that the phytosome complex comprising PFE and PC may enhance bio‐absorption, consistent with findings from prior studies (Jagtap et al. 2023). The strong band at 1612 cm^−1^ in PFE was also observed in NP samples, exhibiting a slight shift likely attributable to a higher wavenumber associated with carbonyl (C=O) groups in PC. A significant difference between PFE and PC was noted at 1343 cm^−1^, related to C=C stretching vibrations of the aromatic ring, which shifted to 1452 cm^−1^ in the PFE‐NP complex. Unique bands corresponding to C—O—C stretching vibrations at 1070 cm^−1^ and C=O bending vibrations at 635 cm^−1^ in PFE were also present in the PFE‐NP complexes, albeit with reduced intensities and slightly shifted wavenumbers. The FTIR spectrum of NP complexes indicated that changes in band intensities and wavenumbers reflect the incorporation of PFE into the nanophytosome structure, corroborating results related to entrapment efficiency (Babazadeh, Ghanbarzadeh, and Hamishehkar 2017; Khosh Manzar et al. 2021; Molaveisi, Shahidi‐Noghabi, and Naji‐Tabasi 2020).

Moreover, in the spectrum, the choline N‐CH in lecithin shifts to a higher frequency in the phytosome spectrum (1038–1098 cm^−1^), with a decrease in intensity suggesting an interaction between lecithin and the extract at the choline level (Rasaee et al. 2014). Overall, FTIR analysis indicates that the encapsulation of PFE primarily occurs through hydrogen bonding. Previous studies also have suggested that phenolic compounds within nano‐phytosomes can be entrapped by PC through stoichiometric interactions, resulting in hydrogen bonds between the OH groups of lecithin and phenolic compounds (Singh et al. 2021). In this research, calcium chloride salt was used to improve the phytosomal system. The addition of calcium chloride salt with the hydroxyl and carboxyl groups in the phytosome structure creates bonds that act as hydrogen bonds. These bonds increase the strength and stability of the phytosome structure.

### 3.7 X‐Ray Analysis of Nanophytosomes Complexes

**FIGURE 6:**
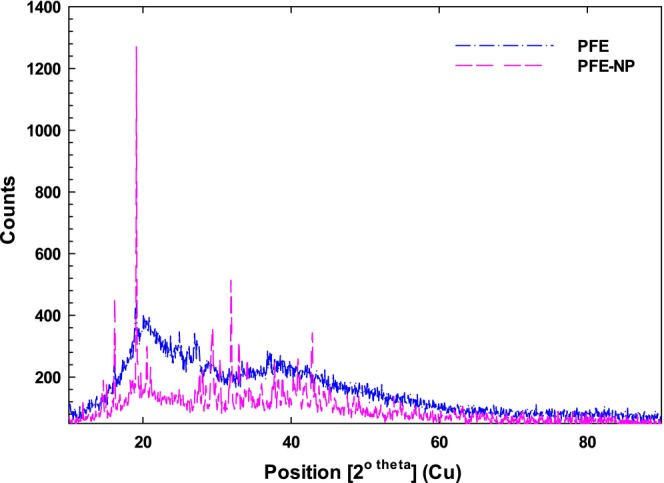
X‐ray diffraction patterns of PFE and PFE‐NP_3_.

X‐ray diffraction (XRD) was employed to investigate the crystallinity and amorphous characteristics of the PFE‐Nano phytosome complex, as depicted in Figure 6. The diffractogram revealed sharp peaks indicative of a crystalline structure, alongside broader peaks suggesting amorphous traits. Specifically, the PFE‐Nanophytosome displayed a crystalline nature with five prominent diffraction peaks at 2θ values of 16°, 19°, 29°, 32°, 33°, and 42°. Notably, peaks around 2θ angles of 10° and 22°, typically associated with protein structures (α‐helix and β‐sheet), were absent, which is further corroborated by FTIR analysis. The PFE diffractogram exhibited a significant peak at 20° (2θ), which may suggest the presence of electrostatic interactions resulting from covalent bonding (Wang et al. 2024). Additionally, broad peaks around 20° in the XRD patterns of the PFE‐Nanophytosomes complex indicate that the interactions between PFE and the nanocarrier involve both hydrogen bonding and electrostatic forces, as confirmed by FTIR results. The XRD pattern of calcium‐reinforced PFE‐Nanophytosomes displayed similar characteristics (Figure 6), reflecting an increase in crystallinity compared to the unmodified complex (Bamgbose et al. 2014).

### 3.8 Thermal Properties of PFE‐NP Complexes

**FIGURE 7:**
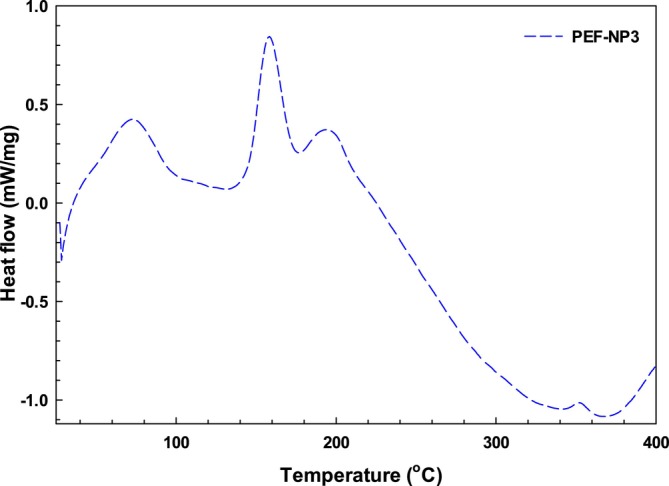
DSC thermogram of nano‐phytosome of PEF‐NP3.

The differential scanning calorimetry (DSC) thermogram for the pomegranate fruit extract (PFE) nano‐phytosome is illustrated in Figure 7. The denaturation temperatures are indicative of protein thermostability, while the enthalpy change (ΔH) reflects the nature of hydrophobic and hydrophilic interactions, as well as the overall compactness of the protein structure. The DSC analysis revealed an endothermic peak for PFE in the range of approximately 307°C–320°C. Additionally, broad endothermic peaks below 70°C were observed, likely corresponding to the evaporation of bound water from the extract. In contrast, soy lecithin exhibited an endothermic peak between 177°C–167°C, suggesting its amorphous characteristics. Interestingly, the endothermic peaks associated with the pomegranate extract were absent in the nano‐phytosome formulation. Furthermore, the expected endothermic peak for calcium chloride, which typically appears in the range of 148°C–165°C, was not detected; only a peak at 48°C–50°C was noted in the phytosome. These observations are consistent with previous studies (Human et al. 2023; Ren et al. 2025). The decomposition enthalpy (ΔH) quantifies the energy required to break down the native structure of the polymer. For PFE and the PFE‐Nanophytosomes complexes, ΔH values were recorded at 249.62 and 331.61 J/g, respectively. This indicates that the cross‐linked complex with calcium chloride necessitates greater thermal energy for decomposition and does so at elevated temperatures compared to its uncross‐linked counterpart (Zhu et al. 2009). Supporting this finding, Timilsena et al. reported denaturation enthalpy values for chia seed protein isolate (CPI), uncross‐linked CPI‐chia seed gum complexes, and cross‐linked CPI‐chia seed gum complexes as 3.2, 13.8, and 16.8 J/g, respectively. Their results demonstrated that cross‐linked complexes exhibit higher enthalpy values than pure polymers, uncross‐linked coacervates, and β‐lactoglobulin‐polysaccharide complexes. Thus, the elevated ΔH values of the PFE‐Nano phytosome complexes indicate their potential as effective shell materials for encapsulating thermally sensitive core substances when compared to individual PFE and phytosomes.

## 4 Conclusion

This study developed enhanced nanophytosomes to improve the stability and bioactive properties of pomegranate fruit extract (PFE) for food and pharmaceutical uses. By utilizing a lipid nanocarrier system with cost‐effective plant compounds and essential nutrients, including phosphatidylcholine (PC) and calcium chloride (CaCl_2_), researchers produced nanoparticles from PFE powder derived from the Raver red seed variety in Kerman, Iran. Using the thin film hydration method, they achieved high encapsulation efficiency, small particle sizes, and low polydispersity indices (PDI). FTIR analysis confirmed that PFE encapsulation occurs mainly through hydrogen bonding, enhancing its antioxidant stability during storage at 4°C for up to 60 days. The study demonstrated that the PFE‐PC‐CaCl_2_ structure improves stability and shelf life, validated by ζ‐potential measurements. The research developed a nano‐colloidal delivery system enhanced with CaCl_2_ salt and pomegranate fruit extract powder, which showed promising industrial applications. Uniform spherical nanoparticles, prepared via thin‐film hydration, showed high EE values, compact size, and narrow PDI. The incorporation of CaCl_2_ enhanced the stability of nanoparticles, and the addition of 2.7 mM CaCl_2_ significantly increased their integrity. The results highlight the nanophytosomes' potential for practical applications in food and pharmaceuticals, emphasizing their effectiveness in preserving PFE's bioactive properties while facilitating controlled release and enhancing storage stability. This research underscores the role of nanotechnology in advancing food and pharmaceutical product development. While the study successfully demonstrated the effectiveness of the squeezing method for extracting phenolic compounds from pomegranates, several limitations should be acknowledged. Firstly, the extraction process may not capture the full range of bioactive compounds present in the fruit, especially those more soluble in organic solvents. Additionally, variations in fruit ripeness and processing conditions could influence the consistency and yield of the bioactive extracts. The study's findings are also limited to the pomegranate varieties used, which may not represent all cultivars. Lastly, further research is needed to evaluate the long‐term stability and bioavailability of the extracted compounds in various food applications. These limitations highlight the need for additional studies to optimize extraction techniques and broaden the understanding of the health benefits of pomegranate‐derived products.

## Author Contributions

**Ramesh Sedighi:** formal analysis (equal), investigation (equal), methodology (equal), writing – original draft (equal). **Ali Rafe:** conceptualization (equal), project administration (equal), supervision (equal), validation (equal), visualization (equal), writing – original draft (equal), writing – review and editing (equal). **Ghadir Rajabzadeh:** conceptualization (equal), supervision (equal), writing – original draft (equal), writing – review and editing (equal). **Abbas Pardakhty:** funding acquisition (equal), methodology (equal), resources (equal), software (equal).

## Conflicts of Interest

The authors declare no conflicts of interest.

## Supporting information


**Figure S1.** Gallic acid standard curve (A), flavonoid standard curve (B), and antioxidant activity (C) ‐ DPPH free radical inhibition.

## Data Availability

Data will be made available on request.
