# Supplementary material for: Development and Characterization of Calcium Ion‐Enhanced Nanophytosomes Encapsulating Pomegranate Fruit Extract
Source: Food Sci Nutr. 2025 Feb 14;13(2):e70032. doi: 10.1002/fsn3.70032 (PMC11828700; doi:10.1002/fsn3.70032)
Supplement: Supplementary file 1 — Figure S1. Gallic acid standard curve (A), flavonoid standard curve (B), and antioxidant activity (C) ‐ DPPH free radical inhibition. [file FSN3-13-e70032-s001.docx]

**Supplementary data**

**Development and Characterization of Calcium Ion-Enhanced Nanophytosomes** **Encapsulating Pomegranate Fruit Extract**

Ramesh Seddighi,^1^ Ali Rafe^1*^, Ghadir Rajabzadeh^2^, Abbas Pardakhty^3^

^1^ Department of Food Physics, Research Institute of Food Science and Technology (RIFST), Mashhad, Iran

^2^ Department of Nanotechnology, Research Institute of Food Science and Technology (RIFST), Mashhad, Iran

^3^ Pharmaceutics Research Center, Neuropharmacology Institute, Kerman University of Medical Sciences, Kerman, Iran

* Corresponding author: Prof. Dr. Ali Rafe

E-mail: [a.rafe@rifst.ac.ir](mailto:a.rafe@rifst.ac.ir)

Orcid id: 0000-0001-7756-9383

**Fig. S1.** Gallic acid standard curve (A), flavonoid standard curve (B), and antioxidant activity (C) - DPPH free radical inhibition.
